# Introducing intrauterine antibiotic infusion as a novel approach in effectively treating chronic endometritis and restoring reproductive dynamics: a randomized pilot study

**DOI:** 10.1038/s41598-021-95072-w

**Published:** 2021-08-02

**Authors:** Konstantinos Pantos, Mara Simopoulou, Evangelos Maziotis, Anna Rapani, Sokratis Grigoriadis, Petroula Tsioulou, Polina Giannelou, Nikolaos Nitsos, Panagiotis Tzonis, Michael Koutsilieris, Konstantinos Sfakianoudis

**Affiliations:** 1 Centre for Human Reproduction, Genesis Athens Clinic, Papanikoli, 15232 Athens, Greece; 2 Department of Physiology, Medical School, National and Kapodistrian University of Athens, 75, Mikras Asias, 11527 Athens, Greece; 3 Assisted Conception Unit, 2nd Department of Obstetrics and Gynecology, Aretaieion Hospital, Medical School, National and Kapodistrian University of Athens, Vasilissis Sofias, 11528 Athens, Greece

**Keywords:** Infertility, Urogenital reproductive disorders

## Abstract

The chronic nature of Chronic Endometritis (CE) along with the challenging management and infertility entailed, call for cutting-edge therapeutic approaches. This study introduces the novel treatment of intrauterine antibiotic infusion (IAI) combined with oral antibiotic administration (OAA), and it assesses respective performance against the gold standard treatment of OAA. Data sourced herein reports on treatment efficiency and fertility restoration for both patients aiming to conceive naturally or via In Vitro fertilization. Eighty CE patients, 40 presenting with recurrent implantation failure, and 40 with recurrent pregnancy loss, were enrolled in the IVF and the natural conception arm respectively. Treatment was subjected to randomization. Effectively treated patients proceeded with either a single IVF cycle or were invited to conceive naturally over a 6-month period. Combination of IAI and OAA provided a statistically significant enhanced effectiveness treatment rate (RR 1.40; 95%CI 1.07–1.82; *p* = 0.01). No statistically significant difference was observed regarding the side-effects rate (RR 1.33; 95%CI 0.80–2.22; *p* = 0.52). No statistically significant difference was observed for either arm regarding live-birth rate. Following an intention-to-treat analysis, employment of IAI corresponds to improved clinical pregnancy rate-albeit not reaching statistical significance. In conclusion, complimentary implementation of IAI could provide a statistically significant enhanced clinical treatment outcome.

## Introduction

An infectious endometrial environment caused by certain pathologies could compromise establishment of the initial interaction between the embryo and the endometrium^1^. Chronic endometritis (CE) is a case of a persistent endometrial inflammation caused by infectious agents namely *Escherichia Coli, Enterococcus faecalis, Streptococcus agalactiae*, Mycoplasma, Ureaplasma, and Chlamydia. The perplexity of managing CE is attributed to its asymptomatic nature rendering a definitive diagnosis rather challenging. The list of potential symptoms is limited and vague while manifestation of the disease may be mild and may not correspond to severity. Symptoms include pain in the pelvic cavity, dispareunia, uterine bleeding, vaginal infections and cystitis, and mild gastro-intestinal discomfort^2^. CE is described as a chronic disorder due to its long duration and persistent nature, with potentially slow progression and complex causality^3^, severely compromising reproductive potential of patients. More conclusive diagnostic criteria are still under investigation and data concerning therapeutic approaches are constantly emerging in literature.

The impaired endometrium is reflected in the poor clinical pregnancy rates observed in cases of CE patients pursuing pregnancy. Concurrently, in the population of infertile patients, CE presents with a wide range of prevalence impressively reaching 60%^4,5^. The catalytical prospective study of Kitaya employed a large cohort of patients showcasing that in fact 33% of women reporting with recurrent implantation failures (RIF) was diagnosed with CE^6^. What is more, an even higher percentage of patients presenting with RIF and CE reaching 66% has been published^4^. Based on these observations, a status of compromised fertility could be an indication to investigate a hitherto undiagnosed and underlying CE, especially when the latter pathology may present as asymptomatic.

Embarking on an antibiotic scheme could potentially treat CE and subsequently address infertility issues^6^. Oral antibiotic administration (OAA) constitutes the gold standard approach for treating CE cases with some studies reporting on a clearance rate of even up to 100%^7^. The deteriorating endometrial environment could be reversed and implantation may be ensued. Nonetheless, effectiveness of treatment of CE is viewed with skepticism since contradicting data is published in regards to the reproductive repercussions following treatment. Certain studies have reported improved clinical results and live birth rates for patients with treated CE pursuing a pregnancy, in comparison to patients with untreated CE^6^. However, others indicate that no differences are documented between the two groups^8^, and therefore-concerning the implantation rate-both schools of thought are represented in literature. Surprisingly, certain studies claim that failing to treat effectively CE does not weigh in particularly with regards to the implantation rates achieved for either group of patients effectively treated or not. The scientific community concurs that it is biologically challenging to decipher how certain published data indicate that patients with untreated CE may even present with a better live-birth rate in comparison to treated cases^8,9^. Irrespectively of the paradox entailed, valid published data should always be included in reaching definitive conclusions leading to statements. Interestingly, it should be noted that, the majority of the studies present with considerable limitations, weaknesses and heterogeneity including the limited number of patients in the study groups, the selected diagnostic tools employed to assess CE prior and following intervention, as well as the fact that they include patients of a variant reproductive potential, which may serve as a confounder in their quest to deliver conclusive results for clinicians.

As reported by Vitagliano the “magnitude of CE diffusion” is both extensive and persistent^10^, and efficiency of the OAA should not be taken for granted. Exploring the scenario of implementing additional approaches to ensure an uninterrupted dialog between the embryo and the endometrium, especially in the case that oral administration fails to be effective, prompt our team of experts to first propose-through a case series report-the approach of intrauterine antibiotic infusion (IAI) as a complementary means towards effectively managing the disease^11^. Emergence of this approach entailing intrauterine infusion of antibiotics was driven by the rational of providing a localized treatment specifically targeting the compromised endometrium, rather than adopting a generalized treatment requiring a systemic response of the patient that is exposed to antibiotic treatment perhaps extending to an administration protocol of prolonged duration. This is achieved by employing a series of antibiotic infusions combined with oral administration in an effort to intensify treatment.

The objective of the present pilot study is to report for the first time on the effects of IAI administration in treating CE and investigate whether it could exert a complimentary enhanced effect when compared to the gold standard oral management. Secondary to that, the present randomized trial aims to report on whether optimal treatment of CE allows fertility restoration. It is evident in literature that despite the established norm to engage on an oral antibiotic treatment to successfully address CE, this approach is not always effective in treating CE to the extent that a status of compromised fertility can be restored. The driver behind design of this study was to introduce a preliminary investigation on an alternative approach which could effectively address and reverse CE, and further restore fertility potential. This approach opens a new line of treatment providing insight on the complementary technique of IAI in combination with oral administration in patients with CE who request to restore reproductive dynamics and pursue a pregnancy following treatment. This novel approach has emerged as a potential rescue call in light of the fact that the chronic nature of the disease and lack of optimal management have not been fully addressed yet. Authors herein aim to contribute further data and evidence to the literature employing a larger population and distinct outcome measures. This study could serve as setting the tone along with expectations that remain to be confirmed or disputed following results from larger Randomized Controlled Trials (RCTs).

## Results

**Figure 1:**
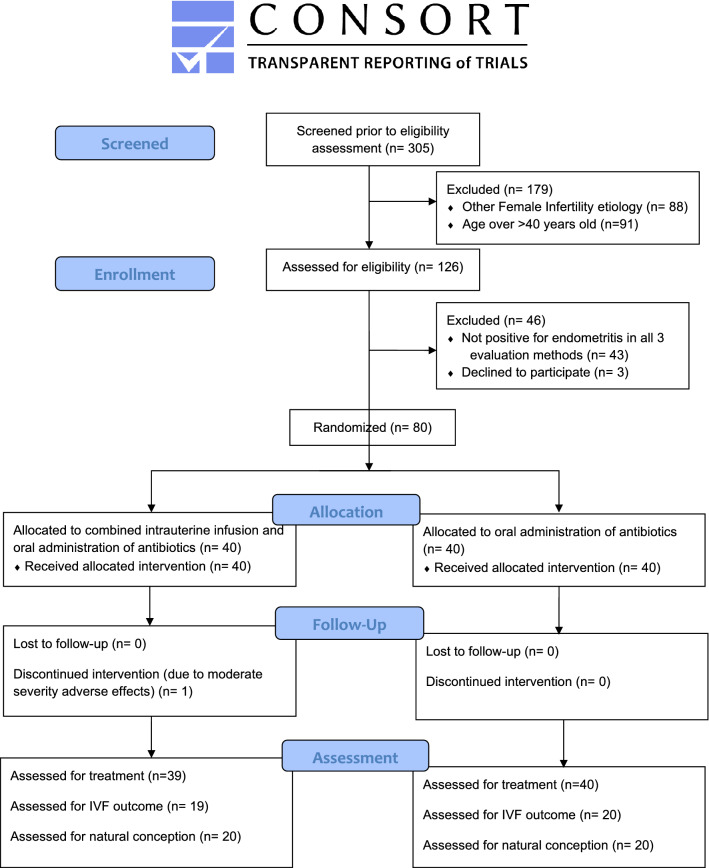
CONSORT diagram presenting the progress throughout the phases entailed in this pilot study, from patients' enrollment to assessment of treatment.

A total of 80 patients were enrolled in both arms of the study. Patients’ age, basal hormonal levels, duration of infertility, previous failed IVF attempts, years of infertility and previous miscarriages did not differ statistically significantly between the groups as presented in Table 1. The pathogens indicated following CE diagnosis for the patients are presented in Table 2. A detailed consort diagram is presented in Fig. 1.

**Table 1:** Mean ± SD of patient characteristics and basic hormonal levels.

	Combination group			Oral administration group		
	IVF	Natural conception	Total cohort	IVF	Natural conception	Total cohort
Age	31.25 ± 1.86	31.65 ± 1.92	31.45 ± 1.97	31.75 ± 1.86	30.90 ± 2.11	31.32 ± 1.91
Years of infertility	6.25 ± 1.48	6.65 ± 1.55	6.45 ± 1.53	6.00 ± 1.59	6.30 ± 1.60	6.15 ± 1.56
Previous pregnancies (gravidity)	0 ± 0	3.00 ± 0.64	1.50 ± 1.58	0 ± 0	2.85 ± 0.67	1.43 ± 1.52
Previous live-births (parity)	0 ± 0	0 ± 0	0 ± 0	0 ± 0	0 ± 0	0 ± 0
Previous failed IVF attempts	3.70 ± 0.65	n/a	n/a	3.85 ± 0.67	n/a	n/a
Recurrent miscarriages	n/a	3.00 ± 0.64	n/a	n/a	2.85 ± 0.67	n/a
FSH	5.80 ± 1.32	5.41 ± 0.91	5.61 ± 1.14	5.50 ± 1.06	5.65 ± 1.15	5.57 ± 1.10
AMH	4.83 ± 1.10	4.59 ± 1.02	4.71 ± 1.05	4.51 ± 0.76	4.63 ± 1.00	4.57 ± 0.88
Progesterone	15.77 ± 1.66	17.25 ± 2.08	16.51 ± 1.89	16.29 ± 1.49	16.50 ± 2.25	16.39 ± 2.00
Days of treatment	29.92 ± 0.28	29.96 ± 0.47	29.93 ± 0.45	14.00 ± 0.00	14.00 ± 0.00	14.00 ± 0.00

**Table 2:** Infectious agents detected by microbiological culture.

Infectious agent	Patients affected
Streptococcus agalactiae	18
Escherichia coli	16
Enterococcus faecalis	15
Ureaplasma	11
Chlamydia	9
Staphylococcus epidermidis	9
Streptococcus gallolyticus	7
Staphylococcus aureus	6
Candida	2

Each patient may present with more than one infectious agent.

The combined administration of antibiotics both orally and via intrauterine infusion provided improved results regarding the number of treated patients when compared to OAA alone (35/40 vs 25/40) increasing the probability of successful treatment by 40% (RR 1.40; 95%CI 1.07–1.82; *p* = 0.01). It should be noted that the number of treatment days was also statistically significantly increased (29.93 vs 15.00; *p* < 0.001) in the combination group.

### IVF arm

A total of 40 patients presenting with RIF were enrolled in the IVF arm of the study. Eighteen out of 20 patients in the combination group were successfully treated. One patient presented with a slight improvement and a decreased inflammation finding, when hysteroscopically assessed, whereas for the remaining patient, the treatment was subject to alteration due to moderate side-effects. Thirteen out of 20 patients in the OAA group were successfully treated, 4 presented with a decreased inflammation finding, and the remaining 3 presented with no change regarding inflammation levels.

Strictly patients that were fully treated proceeded with a single IVF cycle. Number of oocytes, number of fertilized oocytes and number of blastocysts did not differ between the two groups (Table 3). No statistically significant difference was indicated between the combination group (13/18) and the OAA group (9/13) regarding clinical pregnancy (RR 1.04; 95%CI 0.66–1.66; *p* = 0.85). Similarly, no statistically significant difference was indicated between the combination group (13/18) and the OAA group (8/13) regarding live birth (RR 1.17; 95%CI 0.70–1.96; *p* = 0.70). When performing an intention-to-treat (ITT) analysis no statistically significant difference was indicated between the two groups regarding neither clinical pregnancy (RR 1.18; 95%CI 0.71–1.97; *p* = 0.75) nor live birth (RR 1.63; 95%CI 0.87–3.04; *p* = 0.20).

**Table 3:** Embryological data of the IVF arm.

	Combination	Oral administration
Oocytes retrieved	12.11 ± 2.33	12.85 ± 4.56
Fertilized oocytes	11.50 ± 2.28	10.92 ± 3.06
Embryos cleaved	9.00 ± 1.81	9.31 ± 2.35
Blastocysts	7.33 ± 1.28	8.23 ± 1.79
Top-quality blastocysts	3.67 ± 0.77	3.70 ± .1.03

### Natural conception arm

A total of 40 patients were enrolled in the natural conception arm of the study and were invited to conceive naturally within the course of 6 months. Seventeen out of 20 patients in the combination group were fully treated. One patient presented with a decreased inflammation finding, whereas the remaining 2 presented with no change regarding inflammation levels. Twelve out of 20 patients in the OAA group were successfully treated, 4 presented with a decreased inflammation finding, and the remaining 4 presented with no change regarding inflammation levels.

All patients were invited to conceive naturally within the course of 6 months. No statistically significant difference was indicated between the combination group (11/17) and the OAA group (7/12) regarding clinical pregnancy (RR 1.12; 95%CI 0.66–1.66; *p* = 0.85). Similarly, no statistically significant difference was indicated between the combination group (10/17) and the OAA (7/12) regarding live birth (RR 1.00; 95%CI 0.54–1.87; *p* = 0.98). When performing an intention-to-treat (ITT) analysis no statistically significant difference was indicated between the two groups regarding neither clinical pregnancy (RR 1.49; 95%CI 0.80–2.78; *p* = 0.34) nor live birth (RR 1.35; 95%CI 0.73–2.50; *p* = 0.52).

### Side-effects and adverse effects

The side-effects that were recorded during treatment are presented in Table 4. The grading of adverse effects has been performed according to the Common Terminology Criteria for Adverse Effects (CTCAE) (v5.0)^12^. Briefly, according to CTCAE side effects are categorized as mild, if no specific intervention is indicated, as moderate if a pharmaceutical intervention, albeit without hospitalization, was required, as severe if hospitalization was required, as life-threatening or as lethal. Severe, life-threatening or lethal side-effects were not recorded for either of the two treatment groups. Moderate side-effects were reported for one patient in the combination group. Mild side-effects were reported by 6 patients of the combination group and 4 patient of the OAA group When pooling the number of patients with recorded side-effects, no statistically significant difference was observed between the two groups (RR 1.33; 95%CI 0.80–2.22; *p* = 0.52).

**Table 4:** Side-effects and adverse effects reported and categorized.

	Combination group	Oral administration group
**Minimal**		
Drowsiness	2	2
Headache	2	3
*Total patients*	*4*	*3*
**Mild**		
Diarrhea	1	–
Nausea	2	1
*Total patients*	*2*	*1*
**Moderate**		
Abdominal pain	1	–
Vomiting	1	–
Upset stomach	1	–
*Total patients*	*1*	*0*

Each patient may have reported more than one adverse effect.

## Discussion

According to our results, treatment of CE employing a combination of OAA and IAI provided enhanced treatment effects when compared to merely OAA. The OAA group in our study seems to present with a lower effective treatment rate compared to other studies that report data (62.5% vs > 70%). This could be attributed to specific design of the present study. In our study a single treatment cycle was included, compared to possible two or three treatment cycles included in other studies^4^. The effective treatment rate for the first cycle in literature ranged from 27.8 to 92.1%, constituting the 62.5% an above average treatment effectiveness. It should also be highlighted that concerning the present study only patients presenting with negative results for all 3 diagnostic methods namely hysteroscopy, histological examination and microbiological culture, were regarded as fully treated. This may present as another explanation regarding the considerably different effective treatment rates between studies. The side-effects rate did not differ statistically significantly between the two groups, although treatment was altered for one patient from the combination group. This patient was regarded as not treated for the remaining of the study. Interestingly, to our knowledge, no study has reported on side-effects CE treatment to date.

Live-birth rates in both arms of the study did not differ between the two groups (IVF: 72.2% vs 61.5%; Natural Conception: 58.8% vs 58.3%) regarding treated patients. The live-birth rate in the IVF group is in accordance with other studies^4^. It may seem that the live-birth rate in the present study is higher than in others^6,8^. Notably, patients in both Macannany’s and Kitaya’s studies were older than the patients recruited in our study. Live-birth rate originating from natural conception are in accordance to literature. The ITT similarly, did not provide a statistically significant difference (IVF: 65% vs 40%; Natural Conception: 50% vs 35%). It should be mentioned that statistically significant levels may have not been reached due to the small sample size of the study. Evidently, diagnosis and efficient treatment of CE improves live-birth rate following either IVF or natural conception as one would expect. These observations are supported by a recent meta-analysis in the field^13^. Although our results provided no statistically significant difference with regards to live-birth rates between the combination and the OAA groups, this may be attributed purely to the small sample size for each arm of the study. However, it should be highlighted that the combination regime enabled a higher treatment effectiveness. This result alone stands as a significant outcome. Notably, one would expect that this result on treatment effectiveness may in turn ascertain higher clinical pregnancy and live-birth rates something to revisit and reexamine in future larger studies. Larger studies are required in order to conclusively delineate the effectiveness of the combination regime. A limitation in current literature is the lack of reporting on adverse effects. In our study a minor, non-significant increase in the number of patients presenting with adverse effects was observed in the combination group. It is of vital importance that future studies, preferably RCTs, evaluate both the efficacy and the safety of the combination of oral and intrauterine antibiotic administration.

An interesting point of observation in our study was the discrepancy regarding treatment performance and live birth rates between the group of patients engaging in both oral and intrauterine approach versus the OAA group. Although live-birth rates between two groups presented with no statistically significant difference, nonetheless, data on clinical outcomes on treatment performance were indicative of statistically significant enhanced results when oral antibiotic prescription was combined with IAI. Reaching statistical significance despite the limited number of patients enrolled in this study, suggests that the oral antibiotic scheme’s performance may present with limitations with respect to treatment potential while the combination approach of IAI appears to be of superior performance. Implementation of IAI may enhance antibiotic administration performance by acting in a localized fashion amplifying its impact targeting the endometrium. Considering that treatment of CE is a prerequisite to establish a healthy and complication free pregnancy, authors concur that the approach of IAI presents with added benefit, while it certainly merits further larger-scale investigation.

The most recent Delphi consensus^14^ included only hysteroscopy as the diagnostic tool of option, whereas other diagnostic evaluations may further include Polymerase Chain Reaction (PCR) and/or Next Generation Sequencing (NGS). Notably, the authors opted to rely on data stemming from three established diagnostic tools namely hysteroscopy, histological examination and microbiological culture. This design ascertained avoidance of false positive diagnoses during recruitment, and false negative following treatment. Specifically, patients were excluded from the study if they were not evaluated as positive regarding all 3 diagnostic methods, and were considered negative following diagnosis if evaluated as positive for at least one method. The design regarding the inclusion criteria, may have led to the exclusion of patients with CE due to a false negative diagnosis. However, this does not stand as a limitation since this study aims to assess effectiveness of a novel treatment option, and not report on the epidemiological profile corresponding to CE. A possible limitation of the study is that patients should be assessed as negative in all 3 diagnostic evaluations, which may have led to a possible false negative diagnosis and an under-estimation of the treatment effect.

An intriguing point of interest regarding prevalence and treatment as well as relapse of CE is the contribution of the male factor, due to the fact that the disease could manifest as a result of sexually transmitted diseases^15^. As a matter of fact, an aspect that merits investigation is data on the recurrence of the disease, which is not included in the majority of the studies assessing effectiveness of OAA. Diagnosis and treatment of CE concerns the couple and not individually the female partner alone. This may be an aspect that appears as being overlooked in literature, as the focal point of studies remains the female partner. In our study, male partners were instructed to proceed with a semen microbiological screening and respective antibiotic treatment when required. All patients reported that they had proceeded as advised. However, this was not performed as part of the study but was rather a general guideline that hence lacked control. Nonetheless, as this study-similarly to others in literature-includes solely the female partner, non-inclusion of the male partner stands as an issue that should be addressed and presents as a limitation for our study reporting on CE treatment in the context of restoring fertility status. Contribution of the male factor should be accounted for and included in a study when treating couples, especially when contemplating on the effectiveness of a proposed treatment.

Further addressing and highlighting the limitations entailed in the current pilot study, the list of side-effects documented included drowsiness, headache, diarrhea, nausea, abdominal pain, vomiting and upset stomach. Admittedly, reported side-effects raise a concern and indicate that any antibiotic scheme should be administered with caution and it should be coupled by meticulous monitoring of the patients. On the antipode, certain advantages of IAI should be highlighted to orient clinicians critically in assessing this novel approach. The proposed scheme is limited to a one-month period, contrary to the up to three cycle oral treatment proposed by other studies in literature exceeding the 14 days scheme as per guidelines’ suggestion. A shorter time of intervention along with the localized effect of the IAI suggest that the risk of overexposure and development of resistant restrains may be controlled. What is more, in contrary to the huge heterogeneity amongst studies exploring treatment options for CE patients and reporting on their effectiveness, our study was based on a strict three-level evaluation protocol. Following treatment, a re-evaluation should always be performed based on a combination of hysteroscopy, histological and microbiological findings, especially when restoring a receptive endometrium remains as the goal. Due the complex nature of CE and the lack of consensus in managing and addressing it adequately, this study calls out for the need to set a common ground at least for diagnosing this disorder. For instance, it may be of significance to note that studies concluding that CE has been eradicated following oral approach, do not always perform the sampling procedure optimally, which may in turn lead to poor reliability of results^10^.

Taking a step further while practicing caution, the optimal circumstances under which OAA and IAI perform effectively should be fully elucidated prior to introducing this new line of treatment for horizontal use. Duration of treatment, frequency of administration, volume of administrated antibiotic, should be indicated following conduction of respective studies aiming to delineate the optimal protocol of treatment. Further to that, taking into account the chronic aspect of the disease perhaps patients should be categorized accordingly. In turn this may dictate variation in protocol of administration. The scenario of solely implementing an IAI should also be explored and investigated in case that data emerges supporting localized performance in addressing side-effects. Long-term follow-ups should be conducted and are required when a promising novel treatment emerges. Lack of such data constitutes a major limitation when exploring the optimal approach for the clinician who pursues a strategy for effective treatment. Thus, authors refrain from making any conclusive statements, acknowledging the responsibility of recommending novel approaches in clinical practice. The study design of this pilot study set out to primarily report on efficiency of the newly introduced IAI approach when combined with OAA for CE treatment. Restoration of fertility potential and a positive reproductive outcome following a successful treatment was a secondary outcome measure. Results did not indicate a statistically significant improvement with regards to reproductive outcomes. This could be attributed to the small size of the studied population as well as to the fact that it sample size was not calculated to indicate differences with regards to reproductive outcome. Therefore, it is the study design that does not allow for solid conclusions regarding restoration of the compromised fertility status that is associated with a CE diagnosis. To evaluate fertility outcomes a different study design, in terms of power analysis should be conducted in the future, as the combinative administration of antibiotics seems to exert a small-sized effect. However, authors present an incentive for researchers to pursue exploring this option including not just clinical studies but recruiting basic research in the context of translational medicine. “Will further research support the introduction of IAI as a valid therapeutical complementary option in treating CE? And if so should IAI be employed as a tool for all diagnosed with CE or strictly for certain groups of patients for whom OAA appears inadequate?” These questions emerge following results sourced herein. Randomized controlled trials should be the key towards elucidating the role of combining these two practices for the management of CE pathology.

Restoring the reproductive dynamics is an aspect equally essential to treating this disease. Infertility may persist long after the treatment protocol of choice especially in cases where treatment does not require re-evaluation of the patient. The question remains on “how do we move forward” based on the current evidence sourced from literature and emerging from the current pilot study. The concept of OAA along with IAI for patients diagnosed with CE holds potential, albeit the fact that the antibiotics employed are not intended for an intrauterine use constitutes a backpedal in the era of precision medicine.

## Conclusion

As indicated by the results of our study, complimentary implementation of IAI could provide a statistically significant enhanced clinical treatment outcome. The concept of OAA coupled by IAI for patients diagnosed with CE appears to be promising. Undoubtably, the off-label antibiotic use may present as a limitation in the era of precision medicine. Our results may establish preliminary grounds to fuel research focused on a pharmaceutically developed drug appropriate for endometrial administration. A drug designed to target the endometrium while preserving the normal uterine microbiota, could provide mitigated side-effects, results, and long-term successful management of the pathology while improving the reproductive outcome. This approach may serve as the basis for developing a targeted CE treatment of high efficacy. Administration scheme, dosage, and duration represent areas that merit further investigation to ascertain optimal results. Buttressing CE treatment with employment of IAI may extend to establis favorable grounds for achieving a pregnancy for RIF and recurrent pregnancy loss (RPL) patients further burdened reproductively by an underlying CE diagnosis is.

## Materials and methods

The women included in the present study were referred for CE investigation following either RIF in IVF or RPL originating from natural conception, and without any other infertility aetiology. Enrolment of patients was performed from March 2017 till February 2019, in a single private IVF clinic. This study was registered at ClinicalTrials.gov as NCT04447625 (25/6/2020). RIF was defined as at least 3 failed previous IVF attempts employing good quality embryos, and RPL was defined as at least 2 pregnancy losses without a genetic, metabolic, endocrinological, anatomical or male factor aetiology. A further inclusion criterion for all participants was a reproductive history of no previous live-births. Diagnosis of CE was performed following hysteroscopic investigation, endometrial biopsy, along with histological analysis and microbiological culture. Following a positive diagnosis for CE confirmed by all check-point analyses, the patients were divided into the two arms of the study. Patients presenting with RIF that were successfully treated, proceeded with a single IVF cycle. Patients presenting with RPL that were successfully treated, were invited to conceive naturally over the course of six months. Regarding the number of patients included in the study, an accurate power analysis could not be performed as only a case-series has been conducted heretofore, however a small to medium effect size was anticipated. By employing the published empirical models^16^ a total of 20 patients per treatment arm were included. Depending on the original referral being RIF or RPL, patients were randomized and further subdivided into two additional groups according to the scheme of treatment for CE, one receiving OAA and the other receiving a combination of antibiotics administration oral and via intrauterine infusion. Randomization was performed employing a computer-based randomization algorithm for each arm of the study. A 1:1 randomization sequence has been applied. To elaborate on the randomization procedure, patients presenting with RPL were randomized separately from patients presenting with RIF. Thus, two different randomization procedures were conducted to facilitate the two different referral diagnoses. With regards to the diagnostic techniques, histological evaluation and microbiological culture were performed by independent practitioners, who were blinded regarding the results of the hysteroscopic evaluation. Nonetheless, neither the treating physician nor the patients were blinded and no placebo was employed. The embryologists were blinded regarding the treatment protocol employed. The male partners were advised to investigate a possible infection by undergoing a semen culture analysis. In case of testing positive for infection, the same antibiotic regime was advised to be administered. No follow-up evaluation was performed on the male counterpart. Female patients included in this study were considered successfully treated when all three abovementioned diagnostic tools employed provided negative results proving CE was effectively treated. Recruitment of all three diagnostic evaluations was required in the study design in order to exclude any patients presenting with false negative results. Patients were instructed to proceed strictly with the administered treatment, not being subjected to any additional intervention. All patients reported successful compliance with the provided instructions.

All patients were younger than 35 years old, with no other infertility etiology following basic infertility investigation. Further inclusion criteria were FSH and LH levels—evaluated on day 2 of the menstrual cycle—below 12 IU/ml, AMH levels over 1.1 ng/ml, progesterone levels—evaluated on day 21 of the menstrual cycle—between 2 and 25 ng/ml and BMI between 18.5 and 29.9. Women with endometriosis, current or previous cancer diagnosis, auto-immune, genetic or reproductive disorders were excluded. Couples presenting with a reproductive history of pregnancy loss due to genetic abnormalities or requesting PGT-M cycles were similarly excluded. Couples presenting with male factor infertility diagnosis were similarly excluded. Possible male factor infertility was assessed via semen analysis, for patients in both arms of the study.

### Diagnosis of endometritis

The patients were subjected to diagnostic hysteroscopy on days 9–11 of the menstrual cycle, employing a lens-based 3 mm OD mini-telescope, 1058 angle of visual field equipped with a 3.5 mm OD single-flow diagnostic sheath, as previously described^11^. The presence of polypoid endometrium, micropolyps, stromal edema and diffuse hyperaemia were positive evidence regarding the diagnosis of CE. The endometrial biopsy was performed employing a pipelle. In order to minimize contamination risk, following the placement of a vaginal speculum and cleaning external uterine ostium with an iodine solution, the pipelle was inserted under visual control into the uterine cavity ascertaining the avoidance of any contact with vaginal walls. Endometrial samples were diluted into 2 ml of saline and divided into two aliquots, for histological and microbiological analyses. For histological analysis the endometrium samples were fixated employing neutral formalin and paraffin. In order to stain the microsections, hematoxyline and eosin were engaged. CD138 staining was performed and positivity defined the diagnosis of CE. Regarding the microbiological culture, endometrial samples were Gram stained and further placed to agar medium, 5% sheep blood Columbia Agar Base, Chocolate Agar, Mannitol Salt Agar and Mac Conkey Agar (Bio Merieux, Rome, Italy). The samples were incubated for 48 h in 5% CO2 prior to evaluation. For the definitive diagnosis of CE, the patient should be regarded as positive according to all three diagnostic techniques employed.

### Treatment of CE

**Figure 2:**
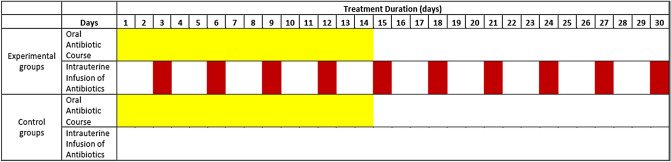
Diagram presenting duration of each treatment approach employed per study group. The control group received oral antibiotic treatment for a duration of 14 days and an administration course of twice daily. Regarding the experimental group involving combined treatment of both oral and Intrauterine administration, commencement of both the oral and intrauterine administration regime was simultaneous. For the first 14 treatment days patient received both oral (twice daily) and intrauterine treatment (one infusion every three days). For the remaining 16 days the patient received only intrauterine infusions to complete the intrauterine infusion administration duration being 30 days including 10 infusions.

Recruited CE patients were divided in two arms depending on the diagnosis they presented with, corresponding to the RIF and RPL arms. Forty patients (n = 40) were included in each of the study’s arms. Then, for both arms, patients were further randomly allocated in two treatment groups, namely the experimental and the control group. Twenty patients were allocated in the experimental group of each arm. An equal number of patients were allocated in the respective control groups. Patients allocated in the experimental groups received a combined course of OAA treatment, concurrently combined with a course of IAI. The total duration of the combined antibiotic treatment was 30 days. In contrast, patients allocated to the control groups received only OAA treatment, for 14 days, according to current guidelines^15^. Description of patient allocation as well as of the course of treatment employed for each of the study’s groups is provided in Table 5 and Fig. 2.

**Table 5:** Description of the study’s groups and respective treatment approaches employed per group.

Pharmaceutical interventions	Recurrent implantation failure arm (n = 40)		Recurrent pregnancy loss arm (n = 40)	
	Experimental group (n = 20)	Control group (n = 20)	Experimental group (n = 20)	Control group (n = 20)
Oral administration of doxycycline (100 mg) Twice a day for 14 days	√	√	√	√
Oral administration of metronidazole (500 mg) Twice a day for 14 days	√	√	√	√
Antibiotic intrauterine infusion of 3 ml ciprofloxacin solution (200 mg/100 ml) 1 infusion every three days. 10 infusions in total in 30 days	√		√

#### Oral antibiotic course

The OAAadministrated for CE treatment included doxycycline administration, which constitutes a wide-spectrum antibiotic effective towards various infectious agents (Park et al., 2016), combined with metronidazole administration, according to the 2015 CDC guidelines for the treatment of pelvic inflammatory disease^15^. Doxycycline was administered at a dosage of 100 mg twice a day for 14 days. Similarly, metronidazole was administered at a dosage of 500 mg twice a day for the same period of time. The total duration of the OAA course was 14 days.

#### Intrauterine antibiotics infusion

Regarding the IAI course, our protocol included infusion of 3 ml of the ciprofloxacin solution for intravenous infusion at a concentration of 200 mg/100 ml. A total of 10 sessions of IAI corresponding to 1 infusion every 3 days for a period of 30 days, were performed for each patient. Antibiotic infusion was performed employing a 23 cm soft Embryo Replacement Catheter as described previously^11^.

### Controlled ovarian stimulation and IVF procedure

Controlled ovarian stimulation was achieved employing the same protocol for all recruited patients. The Controlled Ovarian Stimulation protocol was the standard Gonadotropin-Releasing Hormone (GnRH) long agonist protocol: the initiation of the protocol included administration of 0.1 mg GnRH agonist on day 21 of the previous cycle. Administration of gonadotropin at 300 international units (IU) was employed in a daily dose. When one or more follicles reached a diameter ≥ 17–18 mm, ovulation was triggered employing human chorionic gonadotrophin (hCG) administration. Transvaginal oocyte aspiration was performed 36 h post hCG administration. All patients underwent IVF including a fresh double embryo transfer of blastocyst stage embryos. Embryo grading at the blastocyst stage was performed according to Gardner’s grading system^17^. Blastocysts graded as 4AA, 5AA or 6AA were regarded as top-quality.

### Outcome measures

The primary outcome measures were treatment efficiency as well as the adverse effects rate. Treatment efficiency was defined as a negative CE diagnosis in all 3 diagnostic techniques following treatment. Adverse effects were self-reported by the patients and were categorized according to the CCTAE v5.0. The secondary outcome measures were clinical pregnancy and live-birth either following natural conception or following a single IVF cycle, according to the initial infertility diagnosis.

### Statistics

Data analysis was performed using the R Programming Language for Statistical Purposes. Since the study was designed as a pilot study, the maximum allowed number of participants (n = 20 for each group) were enrolled. Shapiro–Wilk normality test was used in order to check whether the data tested originated from a normally distributed population. Patients’ age, hormonal levels, years of infertility and number of previous failed IVF attempts among the groups were compared employing either t-test or Kruskal–Walis test according to data distribution. The comparison regarding treated patients, clinical pregnancy, live birth and adverse effects rates were performed employing Risk Ratio analysis. Confidence intervals of 95% were calculated for each variable and *p* value < 0.05 was considered statistically significant. Regarding the secondary outcome measures an analysis was performed in patients strictly following successful treatment. RPL patients were invited to conceive naturally, while RIF patients proceeded with IVF. An ITT analysis. was performed solely for the secondary outcome measures including patients that were not successfully treated. In the ITT analysis of the RIF group, the not-fully treated that did not proceed with IVF were recorded as negative in terms of clinical pregnancy and live-birth.

### Study approval

This study was conducted according to the Declaration of Helsinki principles. All patients signed an informed consent regarding their participation to the study. The study was approved by the Centre for Human Reproduction, Genesis Athens Clinic Ethics Board, in Athens, Greece (45/6-2-2017). The consent form informed the patients-regarding the procedure of intrauterine infusion and any possible adverse effects, along with the fact that the respective IVF treatment pursued by the IVF arm of patients would not be subjected to any modification regardless of their decision to participate or not in the study. Moreover, the patients were informed that the participation and subsequent data provided from the cycles would be extracted and analyzed in an anonymous, decrypted method. The patients were invited by the treating physician to decide-in their own time-on the following options: to undergo laparoscopy and participate in the study, to participate in the study without performing laparoscopy and to not participate in the study.

## Data Availability

The data that support the findings of this study are available on request from the corresponding author.
